# Fixation Performance of Bioabsorbable Zn-6Ag Pins for Osteosynthesis

**DOI:** 10.3390/ma15093280

**Published:** 2022-05-03

**Authors:** Salome Hagelstein, Michael Seidenstuecker, Adalbert Kovacs, Roland Barkhoff, Sergej Zankovic

**Affiliations:** 1 G.E.R.N. Tissue Replacement, Regeneration & Neogenesis, Department of Orthopedics and Trauma Surgery, Medical Center-Albert-Ludwigs-University of Freiburg, Faculty of Medicine, Albert-Ludwigs-University of Freiburg, Hugstetter Straße 55, 79106 Freiburg, Germany; michael.seidenstuecker@uniklinik-freiburg.de (M.S.); sergej.zankovic@uniklinik-freiburg.de (S.Z.); 2 Limedion GmbH, Coatings and Surface Analysis, Am Schäferstock 2-4, 68163 Mannheim, Germany; kovacs@limedion.de; 3 Quadralux, Am Schäferstock 2-4, 68163 Mannheim, Germany; r.barkhoff@quadralux.de

**Keywords:** osteosynthesis, fixation performance, shear testing, zinc alloy, bioabsorbable implant

## Abstract

Bioabsorbable implants have become the focus of the latest research for new bone implant materials. With favorable characteristics such as compatible mechanical characteristics, no long-term side effects, and even osteogenesis enhancing properties they seem to be the future of osteosynthesis. Besides these characteristics, they must perform on the same level as traditional implant materials regarding their mechanical support for bone healing. A particular focus in the research for bioabsorbable implants has been on metal alloys, as these have particularly good mechanical properties such as excellent maximum force and high stability. This study focused on the shear strength of new bioabsorbable zinc and magnesium pins in comparison to traditional implants such as K-wires and cancellous bone screws in bone-implant connections. During quasi-static and fatigue loading experiments, magnesium pins (MAGNEZIX, Syntellix AG, Hannover, Germany) and new zinc silver pins (Zn-6Ag) by Limedion (Limedion GmbH., Mannheim, Germany) were compared with conventional osteosynthetic materials. The pins made of the new bioabsorbable alloys withstood the cyclic loads to the same extent as the conventional osteosynthesis materials. In the quasi-static loading, it was shown that the novel Zn-6Ag from Limedion has the same shear strength as the magnesium pin from Syntellix, which is already in clinical use. In addition, the zinc pin showed significantly better shear strength compared to osteosynthesis with K-wires (*p* < 0.05).

## 1. Introduction

In the last few decades, the search for bioabsorbable implants has become a major interest due to their favorable, more bone-like characteristics and the lack of negative long-term effects in comparison to standard implant materials [1,2]. So far, mainly polylactate and polyglucose acid pins and their copolymers are in clinical use [3]. However, they have lower mechanical properties than conventional materials and are therefore only suitable for minimal-load bearing osteosythesis [4,5,6]. Especially with target values for bioabsorbable implants for osteosynthesis with ultimate tensile strengths of 200 to 300 MPa [6,7,8]. Therefore, in recent years, research has increasingly focused on bioabsorbable metals such as magnesium, zinc, and iron alloys, which have significantly better mechanical properties. Especially zinc alloys and magnesium alloys have shown favorable mechanical values as well as good corrosion rates and have been focused on intensively in the latest research [8]. The MAGNEZIX pin, which was brought onto the market in 2015, is the first implant made of bioabsorbable metal available for clinical use [9,10]. However, magnesium implants have shown negative side effects in clinical studies, such as the formation of hypodense areas surrounding the bone in X-ray imaging [11]. These hypodense areas are most likely a result of the production of hydrogen gas during the rapid corrosion of magnesium, as magnesium has a degradation rate of 0.2–2 mm/year [11,12,13]. As the corrosion of magnesium in the biological environment of a human body results in the formation of hydrogen, this can lead to negative effects on bone growth due to additional stress and alkalization of the environment [14]. This can delay the bone healing process in an especially vulnerable phase [15]. 

In comparison, zinc presents a much slower degradation rate of 0.05–0.1 mm/year and, with a standard corrosion potential of −0.76 V, lies between that of magnesium with −2.37 V and iron with −0.44 V [7,15,16,17,18,19]. Furthermore, corrosion in the biological environment of the human body does not result in the production of hydrogen gas and the formation of gas pockets [20,21]. Alloying zinc with silver can significantly change the corrosion rate of silver [19]. For example, Zn-6Ag showed a corrosion rate of 0.11 mm/year, which is similar to corrosion rates of the magnesium alloy used for the MAGNEZIX pin, but yet significantly below the corrosion rates of magnesium [12,22]. These corrosion properties make zinc and even more so Zn-6Ag highly valuable for further research. 

Zinc is also considered to show good biocompatibility while being closely regulated in the human body [18,23]. As it is one of the most abundant trace elements, a daily intake of about 8–11 mg is recommended for adults [24,25]. The maximum total daily intake of zinc is about 40 mg/d [25]. Zinc has been also shown to support cell proliferation and cell function in a dose-dependent manner [26,27]. For example, it has been shown that zinc improved cell migration and cell proliferation in smooth muscle cells and increased mesenchymal stem cell survival and growth rates [28,29]. In addition, differentiation and mineralization of the extracellular matrix increased, and osteogenesis occurred more frequently. Xhao et al. [30] were able to demonstrate through in vivo trials in rabbits that excellent osseointegration had already taken place at the surface of zinc implants in bone after 12 weeks and that zinc promoted the proliferation of bone tissue. 

Silver has long been used as an implant material in humans and has shown very good biocompatibility [31]. Silver occurs naturally as oxides or salts in water and food. The average daily intake through consumption of water ranges from 0.4 to 27 µg, depending on the population [32]. The World Health Organization (WHO) even declared a dose of up to 0.1 mg/l in water to be non-hazardous for health [33]. Silver also has proven to be excellently suited for implantation in bone, with improved regeneration of cancellous and cortical bone [34,35].

The disadvantage of pure zinc is its rather weak mechanical characteristics, with an ultimate tensile strength of only 100–150 MPa [23,36]. Recent research has shown that the initial weaker mechanical properties of pure zinc can be altered favorably with alloying [16,37]. In addition, our research has shown that alloying zinc with silver and titanium can significantly increase the ultimate tensile strength (UTS) [19]. In this study zinc silver titan and zinc, silver alloys reached target values mentioned in the literature for osteosynthetic materials with an ultimate tensile strength of over 200 MPa [6,7]. 

In addition to the mechanical characteristics of the metal alloys, their mechanical performance under clinically relevant conditions is crucial for their evaluation as osteosynthesis materials. For this evaluation, the areas of application in the human body and the load requirements in the area of application play a particularly important role [38,39]. This study focuses on the evaluation of new bioabsorbable metal implants in relation to conventional implants under clinical conditions. In particular, the shear forces of the different osteosynthesis materials were analyzed in comparison to each another. The loads typical after osteotomy in hallux valgus surgery and the mechanical demands on the bone-osteosynthesis connection in post-treatment were used for evaluation. This allows a first clinical assessment of the suitability of the newly developed bioabsorbable implants. In the present work, we compared bioabsorbable novel zinc silver pins (Zn-6Ag) (Limedion GmbH., Mannheim, Germany) and magnesium pins (MAGNEZIX, Syntellix AG, Hannover, Germany) with conventional osteosynthesis materials under dynamic and quasi-static load scenarios for a comparison of maximum force and shear strength in implant-Sawbone connections. 

## 2. Materials and Methods

### 2.1. Materials

This study used shear tests to compare the fixation performance of novel zinc silver (Zn-6Ag) pins in comparison to MAGNEZIX pins (Syntellix AG, Hannover, Germany), cancellous bone screws (DePuy Synthes Companies, Warsaw, IN, USA), and Kirschner wires (K-wires) (Aesculap, Tuttlingen, Germany) in artificial bone. 

The zinc silver pins (Zn-6Ag pins) were manufactured by Limedion (Limedion GmbH., Mannheim, Germany). The Zn-6Ag alloys were cast and refined during hot extrusion with an extrusion rate of 25:1 at 325 °C. Shear testing was conducted using Sawbones 20 PCF open-cell polyurethane foam (1522-12, Sawbones USA, Pacific Research Laboratories Inc., Vashon, WA, United States) as bone substitute material, that was cut into 20 mm × 20 mm × 15 mm squares. 

Testing was conducted with conventional implant materials in comparison to MAGNEZIX pins (Syntellix AG, Hannover, Germany) and the novel Zn-6Ag pin. All implants are listed in Table 1.

### 2.2. Implant Procedure

For testing the implants were inserted into double-joined 20 PCF Sawbones blocks (Sawbones USA, Pacific Research Laboratories Inc., Vashon, WA, USA) vertically. K-wires were inserted directly with a standing drill BF M3 (Arnz Flott GmbH, Remscheid, Germany) at 135 rpm. For the cancellous bone screws and pins, the Sawbones were predrilled with the standing drill at 650 rpm with 2.5 mm or 2.7 mm drill bits respectively. The bone screws were hand-tightened with a maximum torque of 2 Nm with a torque wrench (6003CT, Hermann Zerver GmbH & Co. KG, Remscheid, Germany). The pins were inserted according to the specifications of Syntellix (Syntellix AG, Hannover, Germany) by pre-drilling the blocks and inserting the pins with help of an impactor (impactor 6127.010, Syntellix AG, Hanover, Germany). 

### 2.3. Mechanical Testing

The implants were subjected to generic load scenarios, comparable to general loading conditions after Hallux valgus surgery, and analyzed regarding their quasi-static strength and fatigue strength.

**Figure 1:**
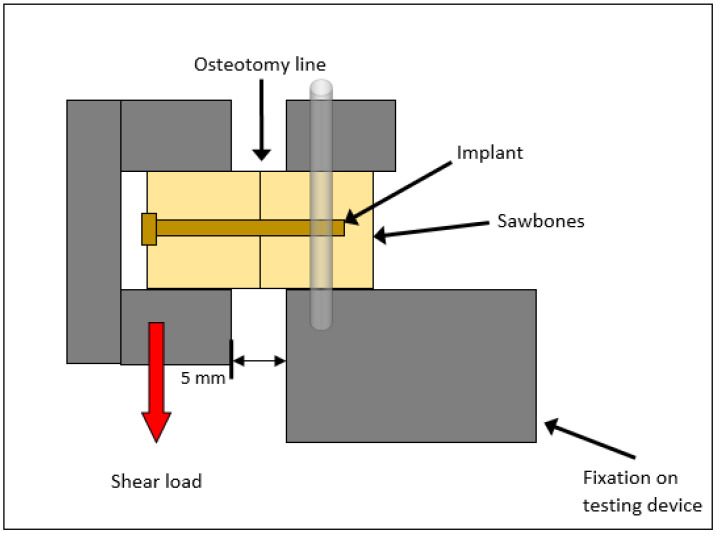
Scheme of the experimental set-up of the shear testing.

Testing was performed with the servo-hydraulic testing machine Amsler HC 10 (ZwickRoell GmbH & Co. KG, Ulm, Germany) with a 10 kN load cell and recorded with the software TestXpert R V1.4.2. (ZwickRoell, GmbH & Co. KG, Ulm, Germany). Special steel holders were manufactured as test holders, each of which clamped a block firmly from three sides. The blocks were clamped horizontally in the Zwick/Roell servo-hydraulic testing machine (see Figure 1).

For fatigue strength, the blocks were subjected to vertical force-controlled sinusoidal loading at 3 Hz. The test started with a preload of 18 N and was loaded cyclically with the forces $F_{max}$ = 30 N and $F_{min}$ = 6 N for 250,000 cycles. This corresponds to an average walking load, with 5000–7000 cycles per day, over a loading period of approximately 6 weeks [40]. Force (N) and displacement (mm) were recorded with 1000 values per second. Failure was defined as a deformation exceeding 2 mm. 

For quasi-static testing, displacement-controlled static tests were performed at a rate of 5 mm/min. The measurement was stopped at the fracture of the sample, which was defined as a shear of 2 mm (with x ≥ 2 mm). Load (N) and displacement (mm) were recorded with 1000 values per second and force-displacement curves were drawn from the data using Origin Pro 2020 (OriginLab, Northampton, MA, USA). Ultimate load ($F_{max}$) was determined from the load versus displacement plot. After the fracture, radiographs of the specimens were taken with the X-ray unit Siremobil-ISO C from Siemens (Siemens AG, Munich, Germany) to determine and document the nature of the failure. 

### 2.4. Statistical Analysis

Statistical analysis was conducted with Microsoft Office Excel^®^, Version 2013 (Microsoft Cooperation, Redmond, WA, USA) and Origin Pro 2020 (OriginLab, Northampton, MA, USA). All quantitative data were given as mean ± standard deviation. Statistical significance was determined with an unpaired one-way analysis of variance (ANOVA) and the significance level was set to *p* < 0.05. 

## 3. Results

### 3.1. Fatigue Testing

Fatigue was tested for all implants. In the cyclic test, all implants used withstood a continuous load of 250,000 cycles. There was no shearing of more than 2 mm in any of the implant-Sawbones connections. 

The maximum shear after 250,000 cycles differed between the implants used. For example, the K-wires had an average displacement of 0.08 mm, the cancellous bone screws reached a maximum shearing of 0.1 mm, and the zinc pins sheared a maximum of 0.09 mm. The greatest change in distance was found in the magnesium pins with an offset of 0.15 mm. However, the differences in the change in distance after cyclic loading were not significant (*p* > 0.18).

### 3.2. Quasi-Static Testing

**Figure 2:**
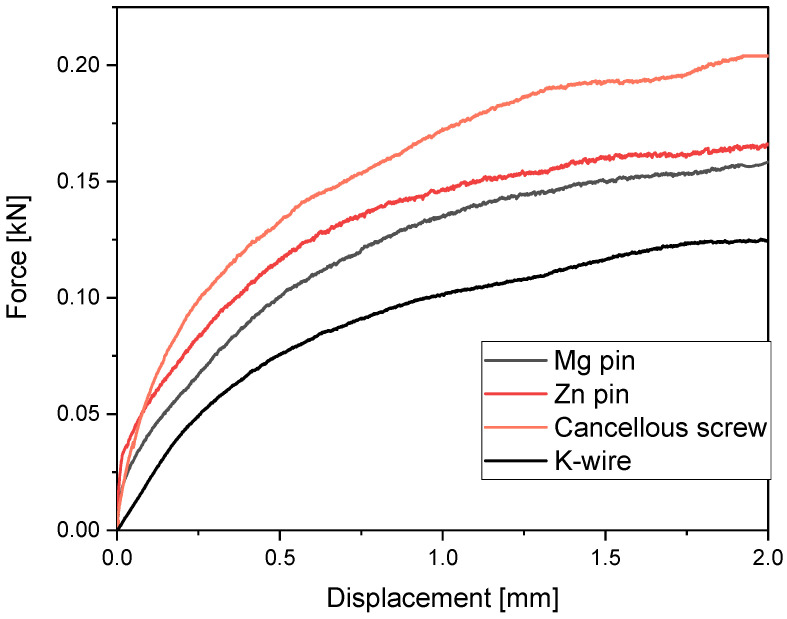
This figure shows the average force-displacement curves of the MAGNEZIX pin, the zinc pin, the cancellous screw, and the K-wire under quasi-static load.

Table 2 lists the maximum forces recorded for the quasi-static testing. The highest failure loads were measured for the cancellous bone screw with 204.84 N. MAGNEZIX pins and zinc pins showed very similar shear strengths with a maximum shear strength of 159.96 N and 166.56 N respectively as seen in Figure 2. 

The shear strengths of MAGNEZIX pins and zinc pins did not differ significantly (*p* = 0.996). However, there was a significant difference in maximum shear strengths between the zinc pins and the K-wires with *p* = 0.046.

**Figure 3:**
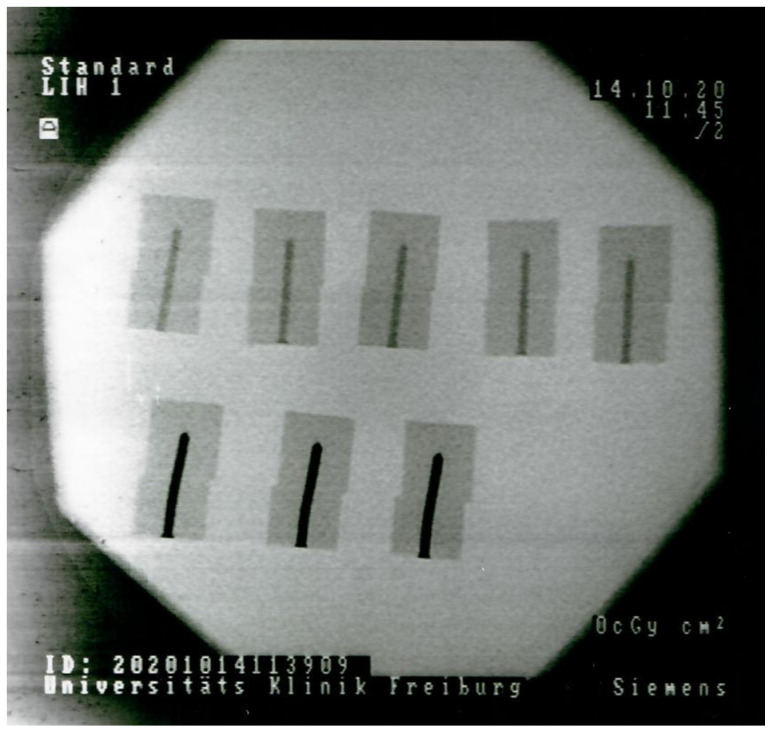
X-ray of the zinc pins (down low) and the MAGNEZIX pins (up high) after quasi-static testing and failure with Siremobil-ISO C from Siemens (Siemens AG, Munich, Germany).

Both the MAGNEZIX pins as well as the zinc pins showed a combined failure of lateral migration and loss of integrity of the Sawbones connection as can be seen in Figure 3. The X-ray image of the implant Sawebones connections after quasi-static loading shows a lateral migration of the Sawebones material as well as a mechanical deformation of the implant.

## 4. Discussion

In the dynamic test, all implant-Sawbones connections withstood the loading conditions. Thus, this study showed that all implants withstood the mechanical demands in the synthetic bone composite that are assumed to occur during a 4 to 6-week healing period after hallux valgus surgery with a forefoot relief shoe [5,40,41]. In the literature, it is assumed that the maximum load in a forefoot relief shoe does not exceed 30 N and that during an average healing period a total of 250,000 cycles with 5000 to 7000 steps a day will occur [40]. These findings also correspond to the results of Augat et al. [5]. They reported no shearing of the Sawbones connections supplied with K-wires under a dynamic load of 4 to 20 N over 250,000 cycles. 

**Figure 4:**
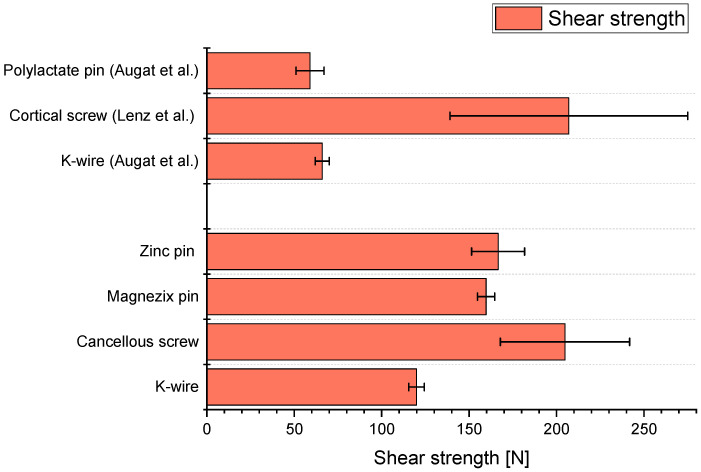
Average maximum shear strengths of the implants measured in this study in comparison to the literature by Augat et al. [5] and Lenz et al. [44] (both with n = 6).

The mean maximum shear strength of the K-wires in the measurements in 20 PCF Sawbones conducted by us was 119.96 N. Augat et al. [5] reported a maximum shear strength of only 66 N for NaCl-conditioned K-wires placed in 20 PCF Sawbones (see Figure 4). Thus, the K-wire connections measured by us showed a greater shear strength. This may be due to the fact that the implants were inserted with different techniques. For example, Downey et al. [42] showed in shear tests with Sawbones with 15 PCF and cancellous screws that the maximum shear strength can be changed by different pre-drilling techniques. Augat et al. [5] did not describe the technique used to insert the K-wires in detail. In our experiments, the Sawbones for the K-wires were not pre-drilled, but the K-wires were inserted directly vertically into the synthetic bones at a low speed of 135 rpm. Another explanation lies in a difference of the acting forces due to a difference in the lever arms. Seebeck et al. [43] showed that the maximum shear strength decreased with a longer lever arm. 

We measured average shear strength of 204 N for the cancellous bone screws in our shear tests. Lenz et al. [44] described maximum shear strengths for 3.5 mm Synthes cortical screws in the human non-osteoporotic bone of 207 N. The same screws reached only 49 N in osteoporotic bone. The measurement in nonosteoporotic bone correlates closely to the measured shear strengths of the cancellous bone screws in this study with an average shear strength of 204 N. Thus, the shear test not only can be validated with literature but also offers the possibility for a first rough assessment of the shear behavior in human nonosteoporotic bone. 

Seebeck et al. [43] measured the shear strengths of Synthes 5 mm LCP screws in human tibiae. They found an average maximum shear strength of 1070 N, which was particularly influenced by the cortical thickness and bone density. The insertion depth was between 21 and 41 mm, depending on the segment height. This is significantly higher than the measured shear strengths in this study, which can be explained by the deeper insertion depth and the different bone compositions. Especially the density and thus the holding force of the surrounding material play a decisive role in determining the maximum shear strength [43,44]. Our testing was conducted with 20 PCF Sawbones, which at 0.32 g/cm³ roughly corresponds to the average density of a metatarsal bone [45,46,47]. Synthetic bones were chosen for the measurements because cadaveric bones have a large variance in bone density, bone shape, and bone structure [48]. This bias in the bones themselves and in the comparison between different bone samples can be eliminated by using synthetic bones. Testing with synthetic bone is already an established and widely performed method of biomechanical testing of osteosynthetic materials on osteosynthesis models and for performing pull-out tests [48,49]. Compared to the values of Seebeck et al. [43], it can be assumed that the differences in the shear strengths occurred due to the different holding forces of the material. 

In this study, the shear strengths of the bioabsorbable magnesium and zinc pins measured did not differ significantly. It can therefore be assumed that the zinc pins inserted into bone withstand similar shear forces as the MAGENZIX pins (Syntellix AG, Hanover, Germany) already in clinical use. The shear strength of implant-Sawbones connections is particularly important for a clinical evaluation of bioabsorbable osteosynthesis materials. Good holding strength and rigidity of the material are especially important for healing after osteosynthesis and are a basic requirement for clinical evaluation [38,39].

In this study during the measurements in artificial bone, failure was a combination of material failure of the implant as well as the Sawbone (see Figure 3). This shows a good connection between the bioabsorbable implant material and the artificial bone material. Both bioabsorbable metal pins showed a maximum shear strength of 160 to 170 N during testing. This compares to much smaller shear strengths of other bioabsorbable pins already in clinical use, e.g., those of the polylactate pins in Augat et al. [5]. Here, the shear strengths of the polylactate pins were reported to be between 50–60 N. Thus, both bioabsorbable metal pins have an excellent average maximum shear strength, not only compared to conventional implant materials but also bioabsorbable pins in clinical use. 

For clinical use, another very important aspect is the altering of the shear strength with progressive corrosion in the biological environment of the human body. Especially, since the altering of mechanical strength during corrosion of bioabsorbable implants plays a huge role during the healing period of bone. Therefore, bioabsorbable zinc alloys need to be further investigated especially for the altering of their mechanical properties under corrosion in test series.

## Figures and Tables

**Table 1:** This table lists the implants, their number, and dimensions used for shear testing.

Implant	Material	Serial No.	n	Dimensions [mm]	
				ø	Length
Cancellous bone screw	Stainless steel	207.030	3	4	30
K-wire	Stainless steel	LX166S	6	1.6	150
MAGNEZIX pin	Mg alloy	1127.030	5	2.7	30
Zinc pin	Zn-6Ag	-	3	2.7	30

**Table 2:** Maximum forces measured in shear testing with mean and standard deviation (SD).

Implant	Mean [N]	SD [N]
K-wire	119.96	4.43
Cancellous bone screw	204.84	36.98
MAGNEZIX pin	159.69	4.94
Zinc pin	166.56	15.14

## Data Availability

The data presented in this article are available on request from the corresponding author.
